# Diverse Bacteriocins Produced by Strains From the Human Milk Microbiota

**DOI:** 10.3389/fmicb.2020.00788

**Published:** 2020-05-19

**Authors:** Angeliki Angelopoulou, Alicja K. Warda, Paula M. O’Connor, Stephen R. Stockdale, Andrey N. Shkoporov, Des Field, Lorraine A. Draper, Catherine Stanton, Colin Hill, R. Paul Ross

**Affiliations:** ^1^School of Microbiology, University College Cork, Cork, Ireland; ^2^APC Microbiome Ireland, University College Cork, Cork, Ireland; ^3^Teagasc Food Research Centre, Moorepark, Fermoy, Ireland

**Keywords:** human milk, bacteriocins, lantibiotics, sactibiotics, antimicrobials, genome mining, antibiotic resistance, human microbiota

## Abstract

Microbial colonization of the infant gut is a convoluted process dependent on numerous contributing factors, including age, mode of delivery and diet among others that has lifelong implication for human health. Breast milk also contains a microbiome which acts as a source of colonizing bacteria for the infant. Here, we demonstrate that human milk harbors a wide diversity of bacteriocin-producing strains with the potential to compete among the developing gut microbiota of the infant. We screened 37 human milk samples and found isolates with antimicrobial activity and distinct cross-immunity profiles. From these isolates, we detected 73 putative gene clusters for bacteriocins of all known sub-classes, including 16 novel prepeptides. More specifically, we detected two novel lantibiotics, four sactibiotics and three class IIa bacteriocins with an unusual modification of the pediocin box that is composed of YDNGI instead of the highly conserved motif YGNGV. Moreover, we identified a novel class IIb bacteriocin, four novel class IIc and two class IId bacteriocins. In conclusion, human milk contains a variety of bacteriocin-producing strains which may provide them a competitive advantage in the colonization of the infant gut and suggests that the milk microbiota is a source of antimicrobial potential.

## Introduction

Human breast milk supplies the newborn with all essential nutrients required for growth (Martin et al., 2016). It also furnishes immunological benefits to the infant and acts as a gut-colonizing bacterial ‘inoculum’ for the infant’s gastro-intestinal (GI) microbiota (Fernández et al., 2013). Human milk is a dynamic milieu that can alter in composition along the stages of lactation (Cabrera-Rubio et al., 2012). Previously, human milk was considered to be sterile, yet many recent studies have provided evidence to the contrary (Heikkilä and Saris, 2003; Collado et al., 2009; Hunt et al., 2011; Mediano et al., 2017). The exact mechanisms of bacterial colonization of milk have yet to be fully elucidated. The skin, the infant oral cavity and even the partner’s microbes are potential sources of bacteria for human milk (Hunt et al., 2011; Kort et al., 2014; Biagi et al., 2017; Ross et al., 2017). Moreover, bacteria of the maternal GI microbiota can spread to the mammary gland through the entero-mammary pathway (Perez et al., 2007).

While international and national health organizations promote exclusive breastfeeding for the first six months of life (WHO, 2000; Abou-Dakn et al., 2010), this may not always be feasible for a variety of reasons (Li et al., 2008). Mastitis is an inflammation of breast tissue and is a common disease that can affect up to 33% of women, often culminating in the discontinuation of breastfeeding (Jiménez et al., 2008; Civardi et al., 2013). Moreover, we have recently found that the incidence of subclinical mastitis could be as high as 38% (Angelopoulou et al., 2020). Major etiological agents of mastitis include *Staphylococcus aureus*, *Staphylococcus epidermidis*, and members of Corynebacteriales. The main standard treatment for the infection is antibiotics (Angelopoulou et al., 2018) although scientists are endeavoring to search for alternative therapies such as bacteriocins (Fernández et al., 2008). These are ribosomally synthesized antimicrobial peptides produced by bacteria that have either broad or narrow range inhibition spectra (Cotter et al., 2013). Bacteriocin producing microbes can be identified via traditional plating methods (O’Sullivan et al., 2019), and more recently through metagenomic *in silico* predictions, using programs such as BAGEL and antiSMASH (Collins et al., 2017; Egan et al., 2018).

Bacteriocins produced by Gram positive bacteria are grouped in two classes, namely class I and class II (Kotelnikova and Gelfand, 2002). The class II bacteriocins may be further subdivided in the pediocin-like (class IIa) bacteriocins, the two-peptide (class IIb) bacteriocins, the cyclic (class IIc) bacteriocins, and the linear, non-pediocin-like (class IId) bacteriocins (Cotter et al., 2005, 2013). Additional bacteriocin subgroups have been also suggested. To date, two class I bacteriocins, lacticin 3147 (Crispie et al., 2005; Klostermann et al., 2009) and nisn (Angelopoulou et al., 2018) have been suggested as alternative treatments for bovine and human mastitis, respectively. They are believed to act as a first line of defense, protecting the host against pathogen invasion (Chikindas et al., 2018). Bacteriocin-producer organisms can impede pathogens from becoming established in a niche by preventing biofilm formation via inhibition of quorum sensing with low level production of bacteriocins (Algburi et al., 2017).

The present study aimed to screen 37 asymptomatic human milk samples for bacteria that produce bacteriocins that target mastitic pathogens with the ultimate aim to identify novel bacteriocin clusters that could provide alternative therapeutic options to counter antibiotic resistance. This study presents discovery and analysis of 16 novel bacteriocin gene clusters.

## Materials and Methods

### Detection and Isolation of Antimicrobial-Producing Human Milk Isolates

Thirty-seven asymptomatic lactating mothers were recruited through Cork University Maternity Hospital to donate breast milk, which was approved by the Cork Clinical Research Ethics Committee and the participants provided written informed consent. Approximately 10 mL aliquots of milk were collected aseptically using sterile gloves in a sterile tube with the first few drops (∼500 μL) being discarded. The nipples and the mammary areola had been cleaned with sterile alcohol-free aqueous solution moist wipes (Ted Kelleher, First Aid & Hygiene Supplies Ltd, Macroom, Ireland) in order to avoid contaminating the milk with skin-colonizing bacteria. Samples were stored below 4°C overnight until they were further processed. Aliquots of 1 mL of milk were cultivated within 24 h of donation. Aliquots of milk sample, 1 mL, were mixed with 9 mL of maximum recovery diluent (MRD; Oxoid, Basingstoke, United Kingdom) to make an initial 10^–1^ dilution. Serial dilutions were enumerated by the spread plate method in duplicate onto: (a) Blood agar base (Oxoid, Basingstoke, United Kingdom) supplemented with 7% v/v defibrinated sheep blood (Cruinn Diagnostics, Dublin, Ireland) and incubated at 37°C for 48 h aerobically; which is a non-selective medium and (b) Baird Parker agar (Oxoid) supplemented with Egg Yolk Tellurite Emulsion (Oxoid) and incubated at 37°C for 48 h aerobically; which selects for staphylococci. Blood agar and Baird Parker plates with separate colonies were replicated in three clean Blood agar and Baird Parker plates, respectively using a replicator stamp (Karl Roth, Karsruhe, Germany). Replicated plates were incubated at 37°C, aerobically for 48 h. The day after the 48 h incubation, plates were exposed to UV light for 45 min and then overlaid with Brain Heart Infusion (BHI) sloppy (Oxoid) containing 0.75% agar seeded with: (a) 0.25% of an overnight culture of *S. aureus* APC 3759 and grown aerobically overnight at 37°C; (b) 0.25% of an overnight culture of *M. luteus* DSM 1790 and grown aerobically overnight at 37°C and (c) 0.25% of an overnight culture of *P. aeruginosa* PA01 and grown aerobically overnight at 30°C. In total, 174 colonies exhibited antimicrobial activity against the tested strains and were grown overnight in BHI, streaked for purity and stocked in 40% glycerol in two 96-well plates which were stored at −80°C for further characterization.

### Cross-Immunity

To determine the relatedness of the bacteriocins produced by the strains, cross-immunity assays were performed during which the producing strains were tested against each other by conducting spot bioassays using each producing strain as a producer and also as a target strain. More precisely, the two 96-well plates were stamped using a replica platter (Sigma-Aldrich, Germany) onto clean Tryptic Soy Agar (TSA) plates or Mueller Hinton (MH; Oxoid) plates; the plates were incubated aerobically overnight at 37°C, before UV irradiation for 45 min. These were then overlaid with the same media (0.75% w/v agar seeded with 0.25% of an overnight culture of the producing strains) and grown aerobically overnight. Cross-immunity was performed for each of the two 96-well plates separately. Strains with a distinct profile from the two 96-well plates were merged into one and cross-immunity was repeated as described-above leading to 80 strains with a distinct profile (Supplementary Table S1). The 80 strains were stored individually.

### 16S rRNA Sequencing

Colony PCR was performed on antimicrobial-producing strains with a distinct cross-immunity profile. Cells were lysed in 10% Igepal 630 (Sigma-Aldrich, Germany) at 95°C for 10 min. PCR was performed in a total volume of 25 μL using 10 μL of Phusion Green Hot Start II High Fidelity PCR master mix (Thermo Fisher Scientific), 10 μL of PCR-grade water, 1.5 μL of the non-specific primers 27F and 1495R (primer stocks at 0.1 ng μL^–1^; Sigma-Aldrich) and 2 μL of DNA template from lysed cells. Amplification was carried out with reaction conditions as follows: initial denaturation at 98°C for 30 s, followed by 35 cycles of 98°C for 10 s, annealing at 55°C for 30 s and elongation at 72°C for 30 s with a final extension step at 72°C for 10 min.

To confirm species, degenerate primers targeting the *dnaJ* gene (Shah et al., 2007) forward primer, 5′-GCCAAAAGA GACTATTATGA-3′, and reverse primer, 5′-ATTGYTTACC YGTTTGTGTACC-3′, were used for the PCR reactions, following conditions described by Shah et al. (2007).

Five microliters of the resulting amplicons from each reaction were electrophoresed in a 1.5% (w/v) agarose gel. A GeneGenius Imaging System (Syngene, Cambridge, United Kingdom) was used for visualization. The PCR products were purified using the GeneJet Gel Extraction Kit (Thermo Fischer Scientific, Waltham, MA, United States). DNA sequencing of the forward strand was performed by Source BioScience (Tramore, Ireland). The resulting sequences were used for searching sequences deposited in the GenBank database using BLAST^1^ and the identity of the isolates was determined on the basis of the highest scores (≥98%).

### Spectrum of Inhibition

The spectrum of inhibition was completed on overnight cultures of 29 antimicrobial-producing isolates with a distinct immunity profile using 29 distinct indicator strains by a spot bioassay as described above with small modifications. More precisely, 5 μL of an overnight culture of the 29 antimicrobial-producing isolates were spotted on a clean TSA plate and incubated aerobically overnight at 37°C, before UV irradiation for 45 min. The rest of the procedure was performed as described above. The indicator strains employed to detect antimicrobial activity and their optimal growth conditions are listed in Table 1. Anaerocult A gas packs (Merck, Darmstadt, Germany) were used to generate anaerobic conditions where necessary for a selection of the indicator strains used in the study. Zone size was measured as follows: diameter of zone – diameter of spot in millimeters. The experiment was performed in three independent replicates.

**TABLE 1:** Growth conditions for the indicator strains used in this study.

**Growth conditions**				

**Species**	**Strain**	**Temperature (°C)**	**Atmosphere**	**Growth media**
*Listeria monocytogenes*	L028	37	Aerobic	BHI, TSB, MH
	ATCC 33007			
	EDG-e			
*Bacillus cereus*	DPC 6087	37	Aerobic	BHI, TSB, MH
*B. subtilis*	NCTC 10073	37	Aerobic	BHI, TSB, MH
*Lactococcus lactis* subsp. *cremoris*	HP	30	Aerobic	GM17
*E. faecium*	DPC 3675	37	Aerobic	BHI, TSB, MH
*E. faecalis*	ATCC 29200	37	Aerobic	BHI, TSB, MH
	DPC 5152			
	DSM 2570			
	VRE V583			
*S. aureus*	APC 3759	37	Aerobic	BHI, TSB, MH
	DPC 5243			
	Newman			
	DPC 7673			
	RF122			
*M. luteus*	DSM 1790	37	Anaerobic	BHI, TSB, MH
*P. aeruginosa*	PA01	30	Aerobic	BHI, TSB, MH
MRSA	BSAC ST528	37	Aerobic	BHI, TSB, MH
	BSAC ST530			
*Escherichia coli*	MG 1655	37	Aerobic	LB
	0157			
*St. agalactiae*	ATCC 13813	37	Aerobic	TSB, MH
*St. uberis*	DPC 5344	37	Aerobic	TSB, MH
*C. kroppenstedtii*	APC 3845	37	Anaerobic	TSB, MH
*C. tuberculostearicum*	APC 3844	37	Anaerobic	TSB, MH
*Cutibacterium avidum*	APC 3846	37	Aerobic	TSB, MH
*St. salivarius*	APC 3843	37	Anaerobic	TSB, MH
*St. pyogenes*	DPC 6992	37	Anaerobic	TSB, MH
*Lactobacillus delbrueckii* subsp. *bulgaricus*	LMG 6901	37	Anaerobic	MRS

*ATCC, American Type Culture Collection; APC, APC Microbiome Ireland Culture Collection; DPC, Teagasc Culture Collection; LMG, Laboratorium voor Microbiologie, Universiteit Gent, Belgium; NCTC, National Collection of Type Cultures, Public Health England; BSAC, British Society for Antimicrobial Chemotherapy; DSM, Leibniz Institute DSMZ – German Collection of Microorganisms and Cells Cultures.*

### DNA Extraction and Sequencing

Genomic DNA was extracted from the 79 strains (with the exception of *S. lugdunensis* APC 3758) using the DNeasy Blood and Tissue kit (Qiagen, Hilden, Germany) with slight modifications. For Gram positive bacteria, 2 mL of an overnight culture (TSB; 1% inoculum) were centrifuged at 18,078 *g* for 10 min and the supernatant was discarded. The cell pellet was resuspended in 180 μL of enzymatic lysis buffer and was incubated for 2 h at 37°C. The remaining steps were performed according to the original protocol. For the Gram-negative isolate *P. protegens* APC 3760, 2 mL of an overnight culture (TSB; 1% inoculum) were centrifuged at 18,078 *g* for 10 min and the supernatant was discarded. The cell pellet was resuspended in 180 μL buffer ATL with the remaining steps being performed according to the original protocol. Genomic DNA was quantified using a Qubit dsDNA HS Assay Kit (Invitrogen, Thermo Fisher Scientific, Waltham, MA, United States). Apart from *S. lugdunensis* APC 3758, the remaining 79 strains were subjected to library preparation using the Nextera XT DNA Library Preparation Kit (Illumina, San Diego, CA, United States) and bead-based normalization following the standard manufacturer’s protocol. Ready-to-load libraries were sequenced using a proprietary modified protocol using 2 × 300 bp paired-end chemistry on an Illumina HiSeq 2500 platform (Illumina) at GATC Biotech AG, Germany.

Cells of *S. lugdunensis* APC 3758 were grown to mid-log phase in Tryptic Soy broth (TSB) and centrifuged at 2,400 *g* for 20 min. A 600 mg pellet of cells was then snap frozen by placing the centrifuge tube into ethanol which had been previously cooled for 24 h to −80°C. Chromosomal DNA was isolated by commercial sequence provider GATC Biotech, Ltd (Kostanz, Germany). Single-molecule real-time (SMRT) sequencing was performed on Pacific Biosciences RS II sequencing platform (executed by GATC Biotech, Ltd.).

The rapid sequencing kit (SQK-RAD004, Oxford Nanopore Technologies) was also used to prepare the *P. protegens* APC 3760 DNA library according to the manufacturer’s instructions. *P. protegens* APC 3760 DNA was sequenced on a MinION device using R9.4 flow cell. The run duration was 24 h. Raw data was processed and basecalled using ONT Albacore Sequencing Pipeline Software (version 2.3.3).

### Genome Assembly and Annotation

Following sequencing, Illumina adaptors were removed from fastq reads using CutAdapt (version 1.9.1; Martin, 2011). Paired-end adaptor-free reads were trimmed using Trimmomatic (version 0.32; Bolger et al., 2014) to a Phred score of 30 across a 4 bp sliding window. Surviving reads less than 70 bp were discarded. The final quality of reads was assessed using FastQC (version 0.11.5)^2^. Finally, both paired and unpaired Illumina reads were assembled using SPAdes (version 3.10.0; Nurk et al., 2017). Assembly of complete *S. lugdunensis* APC 3758 genome from raw Pacific Biosciences SMRT reads was done using Flye (version 2.4.1). Hybrid draft assembly of *P. protegens* APC 3760 genome from trimmed Illumina HiSeq and raw ONT MinION reads was performed using SPAdes in ‘careful’ mode. Annotation of the genomes was provided by the SEED viewer and the RAST annotation server (Overbeek et al., 2014).

### Bacteriocin *in silico* Identification

The bacteriocin mining tool BAGEL3 (van Heel et al., 2013) was used to identify putative bacteriocin operons in combination with antiSMASH5 (Blin et al., 2019) which detects secondary metabolites. The genome visualization tool CLC Sequence viewer 8.0 (CLC Bio, Qiagen, Aarhus, Denmark) was subsequently used for manual analysis of the bacterial genomes. To determine the degree of novelty in the bacteriocins identified by BAGEL3 and antiSMASH5, BLASTP (Altschul et al., 1990) searches were performed for each putative bacteriocin peptide against those identified in the BAGEL and antiSMASH screen. The levels of identity described in this study are derived from MUSCLE. Structural peptides were aligned using the Multiple Sequence Alignment (MSA) tool MUSCLE (Edgar, 2004) and then visualized using Jalview (Waterhouse et al., 2009). Novelty was defined when there was a difference of two or more amino acids between the predicted bacteriocin and the one which was previously discovered.

## Results

### Isolation of Potential Antimicrobial-Producing Human Milk Isolates

Thirty-seven lactating mothers were recruited who agreed to donate milk samples. Approximately 5,100 colonies isolated from these samples were screened against three indicator organisms; *S. aureus* APC 3759, *Micrococcus luteus* DSM 1790 and *Pseudomonas aeruginosa* PA01. *S. aureus* APC 3759 was chosen as an indicator microorganism as it is not a bacteriocin producer and is just a representative strain. *P. aeruginosa* PA01 is a Gram-negative bacterial indicator whereas *M. luteus* DSM 1790 was selected based on its use to screen for antimicrobial activity (Janek et al., 2016; Wayah and Philip, 2018). No strain displayed inhibitory activity against *P. aeruginosa* PA01, but 174 potential antimicrobial-producing isolates were active against at least one of the other two indicators. Isolates with antimicrobial activity were identified in 25 out of 37 milk (67.6%) samples (data not shown).

One of the essential features of bacteriocin-producing bacteria is the requirement for immunity genes, which protect producers from the antimicrobial action of the bacteriocin they produce. Consequently, strains showing the same immunity pattern are likely to have the ability to produce the same bacteriocin(s). Based on the cross-immunity assay from 174 potential antimicrobial-producing isolates, 80 distinct profiles were observed by eliminating strains that displayed cross-immunity and consequently suspected to produce the same or a very similar bacteriocin (Supplementary Table S1).

16S rRNA amplicon sequencing of the 80 antimicrobial-producing isolates revealed one Gram-negative bacterium namely *Pseudomonas protegens*, 47 *S. epidermidis isolates*, one *S. hominis*, one *S. lugdunensis*, 18 *S. aureus* isolates, one *Enterococcus faecalis*, and 11 *E. faecium* isolates. Subsequently, the genomes of the 80 identified isolates were sequenced to enable genome-mining to identify the antimicrobial encoding genes and their potential novelty.

### Spectrum of Inhibition

**FIGURE 1:**
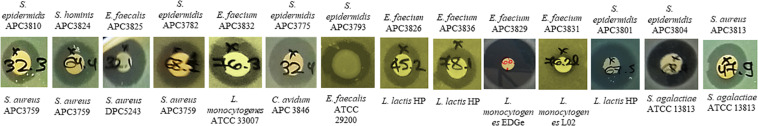
Representative zones of inhibition from a selection of the 29 antimicrobial producers that demonstrated the strongest inhibition against *S. aureus* APC 3759 and *M. luteus* DSM 1790 against a selection of the 29 indicator strains.

We selected 29 of the 80 strains that exhibited the strongest inhibitory activity against the indicator organisms *S. aureus* APC 3759 and *M. luteus* DSM 1790 (data not shown) for examination of their inhibition spectra. Table 2 shows the inhibition spectra of a panel of 29 antimicrobial producers against 29 indicators including known human mastitis pathogens such as *S. aureus* (Delgado et al., 2011), Methicillin Resistant *S. aureus* (MRSA) (Holmes and Zadoks, 2011), *Corynebacterium kroppenstedtii* (Wong et al., 2017) and *C. tuberculostearicum* (Paviour et al., 2002). The detected zones of inhibition could be attributed to any of the antimicrobial(s) produced by each of the tested strains. The antimicrobial produced by *E. faecalis* APC 3825 demonstrated the broadest spectrum of activity, inhibiting 19 of the indicator strains including vancomycin resistant *E. faecalis* V583, *S. aureus* APC 3759, *S. aureus* Newman, *S. aureus* DPC 5243, *St. pyogenes* DPC 6992, *C. tuberculostearicum* APC 3844 and the bovine mastitis pathogens *St. agalactiae* ATCC 13813 and *St. uberis* DPC 5344. *S. epidermidis* APC 3806, *S. epidermidis* APC 3804, and *S. epidermidis* APC 3782 inhibited 16, 15, and 14 of the indicators, respectively. All three of these *S. epidermidis* strains were isolated from the same donor (Table 3) yet their inhibition profile differed. *S. aureus* APC 3813 and *S. epidermidis* APC 3810 exhibited the narrowest inhibition spectra – inhibiting only three of the tested indicators. *S. lugdunensis* APC 3758 and *P. protegens* APC 3760 displayed inhibitory activity against the tested *S. aureus* strains, including the two MRSA strains, namely ST528 and ST530. Twenty-three of 29 strains inhibited the growth of *S. aureus* strains tested with only two impeding the specific MRSA strains tested. Representative zones of inhibition from a selection of the 29 potential bacteriocin producers are presented in Figure 1. None of the 29 potential bacteriocin producers inhibited *Corynebacterium kroppenstedtii* APC 3845, *Bacillus cereus* DPC 6087, *B. subtilis* NCTC 10073, *E. coli* MG1655 or *E. coli* O157.

**TABLE 2:** Spectrum of inhibition of potential bacteriocin-producing human milk isolates against indicator strains by spot bioassays.

**Isolates ↓/Indi cator →**	***L. mono cyto genes* L028**	***L. mono cyto genes* ATCC 33007**	***L. mono cyto genes* EDGe**	***L. lactis* HP**	***M. lut eus* DSM 1790**	***E. fae cium* DPC 3675**	***E. faec alis* ATCC 29200**	***E. faec alis* DPC 5152**	***E. faec alis* DSM 2570**	***E. faec alis* VRE V583**	***S. aur eus* APC 3759**	***S. aur eus* DPC 5243**	***S. aur eus* New man**	***S. aur eus* MRSA ST528**	***S. aur eus* MRSA ST530**	***S. aur eus* DPC 7673**	***S. aur eus* RF122**	***St. agala ctiae* ATCC 13813**	***St. ube ris* ATCC 5344**	***C. tuber culos tear icum* APC 3844**	***C. avi dum* APC 3846**	***St. saliv arius* APC 3843**	***St. pyo genes* DPC 6992**	***L. bulg aricus* LMG 6901**
***E.faecalis* APC 3825**	1.5± 0.3			11.5± 0.6	9.9± 0.2	10.6± 0.3	11.7± 0.5	2.5± 0.7	1.4± 0.4	7.6± 0.9	9.9± 0.2	5.3± 0.5	9.9± 0.2					7.4± 0.2	12.7± 0.4	1.4± 0.4	5.3± 0.5	7.6± 0.9	7.9± 0.3	
***E.faecium***																								
**APC 3835**	1.7± 0.2	5.1± 0.6	6.5± 0.6	4.9± 0.4			2.3± 0.3				2.1± 0.1							4.1± 0.4						
**APC 3832**	1.0± 0.2	4.7± 0.3	7.0± 0.5	5.3± 0.2		2.2± 0.3					2.5± 0.3													
**APC 3836**	1.5± 0.2	5.3± 0.5	7.0± 0.6	5.4± 0.2		2.1± 0.3	2.8± 0.2				2.7± 0.4							2.0± 0.6						
**APC 3826**	1.3± 0.2	5.8± 0.5	9.4± 0.5	5.2± 0.2		2.4± 0.2	2.0± 0.2				2.8± 0.6							2.0± 0.6						
**APC 3830**	1.0± 0.2	4.9± 0.5	7.2± 0.7	1.5± 0.4			2.8± 0.2				3.1± 0.2													
**APC 3837**	1.2± 0.2	5.0± 0.4	6.7± 0.4	6.3± 0.5							3.4± 0.1							1.9± 0.6						
**APC 3831**	1.1± 0.1	5.1± 0.4	6.2± 0.4	5.2± 0.6							4.5± 0.2							1.8± 0.6						
**APC 3833**		4.6± 0.4	6.5± 0.5	4.4± 0.3				4.4± 0.3			5.1± 0.3							4.2± 0.3						
**APC 3829**	2.2± 0.2		6.0± 0.4		4.2± 0.3						5.3± 0.2													
***S. epider midis***																								
**APC 3793**					7.8± 0.3	9.7± 0.6	5.2± 0.2	6.0± 0.3		6.4± 0.4								5.8± 0.4						
**APC 3803**		3.0± 0.8		8.8± 0.5	4.0± 0.3	6.2± 0.3	4.1± 0.3	5.7± 0.4		5.3± 0.4								6.2± 0.4	5.9± 0.3					
**APC 3801**				13.6± 0.1	8.7± 0.4	8.7± 0.3	5.6± 0.4			6.4± 0.2		9.0± 0.5						6.4± 0.4	6.1± 0.3					
**APC 3782**	7.8± 0.5		6.0± 0.2		11.7± 0.5	12.6± 0.5	7.5± 0.3	12.0± 0.5		9.1± 0.4	4.5± 0.4					5.0± 0.3		12.0± 0.6	8.6± 0.4		7.4± 0.1	6.1± 0.8	7,4± 0.4	
**APC 3804**	5.0± 0.2		6.0± 0.2	17.3± 0.2	11.5± 0.4	5.8± 0.8	12.0± 0.4	11.9± 0.6		9.7± 0.5	6.0± 0.4					5.0± 0.4		11.0± 0.6	9.7± 0.3		9.0± 0.4	9.9± 0.5	6.6± 0.3	
**APC 3806**	7.4± 0.2	1.5± 0.5		16.5± 0.7	10.3± 0.4	11.7± 0.3	8.6± 0.3	12.8± 0.2		9.2± 0.4	6.0± 0.2	9.1± 0.7				5.0± 0.2		10.9± 0.4	11.3± 0.4		10.3± 0.1	6.5± 0.8	5.9± 0.3	
**APC 3810**				5,6± 0.4							4.1± 0.2													7.9± 0.5
**APC 3775**				15.5± 0.2	7.0± 0.3	9.2± 0.3	5.5± 0.2	6.2± 0.2	7.7± 0.9	6.8± 0.4								5.8± 0.4	5.4± 0.3	11.1± 0.4		9.9± 0.4	6.1± 0.3	
***S. aureus***																								
**APC 3774**	3.6± 0.3			17.4± 0.3	14.3± 0.4	14.1± 0.5	6.5± 0.3	6.6± 0.2	8.9± 0.9	7.3± 0.4		10.3± 0.4						8.5± 0.3	6.3± 0.2	12.2± 0.5				
**APC 3787**	6.0± 0.3				7.9± 0.5	8.9± 0.4	6.7± 0.4	5.2± 0.3		6.0± 0.1								6.3± 0.3	6.3± 0.2					
**APC 3887**				20.0± 0.2	8.1± 0.3	7.6± 0.6	5.0± 0.3					11.5± 0.3												7.0± 0.3
**APC 3809**				9.7± 0.6	5,3± 0.6							8.7± 0.2						3.8± 0.3						7.4± 0.4
**APC 3823**				15.1± 0.2	9,1± 0.4							10.7± 0.2						2.7± 0.3						9.5± 0.3
**APC 3813**				9.6± 0.6	5.8± 0.3													5.2± 0.3						
**APC 3822**				3.9± 0.4	4.6± 0.5							5.3± 0.2						4.7± 0.5						
**APC 3812**				4,3± 0.4	4.8± 0.4													5.2± 0.4						3.1± 0.6
***S. hominis* APC 3824**						13.1± 0.5	7.8± 0.1			3.8± 0.6	4.7± 0.2													
***S. lugdun ensis* APC 3758**											3.0± 0.2	3.0± 0.1	3.0± 0.2	3.1± 0.2	3.0± 0.2		2.9± 0.2						2.1± 0.2	
***P. prote gens* APC 3760**											4.5± 0.3	4.0± 0.2	4.1± 0.3	3.5± 0.2	3.4± 0.4	2.9± 0.2	3.0± 0.2							

*The numbers indicate the size of the inhibition zone with standard deviation. The zone size was measured as follows: diameter of zone – diameter of spot in millimeters. The experiment was performed in three independent replicates. None of the 29 potential bacteriocin producers inhibited *C. kroppenstedtii* APC 3845, *B. cereus* DPC 6087, *B. subtilis* NCTC 10073, *E. coli* MG1655 or *E. coli* O157.*

**TABLE 3:** Antimicrobial prediction by BAGEL3 and antiSMASH5 for the 80 bacterial isolates with distinct cross-immunity profile.

**Donors**	**Strains**	**Bacteriocin**		**Delta-lysin**	**Siderophore**	**NRPS**	**CDPS**	**Phosphonate**	**Others**
		***BAGEL3***	***antiSmash5***	***BAGEL3***	***antiSmash5***	***antiSmash5***	***antiSmash5***	***antiSMASH5***	***antiSMASH5***
**H1**	***S. aureus* APC 3809**	Class IId	–	2	Aureusimine	–	–	Terpene	
	***S. epidermidis* APC 3785**	Sactibiotic	–	–	Staphyloferrin	1	–	–	–
	***S. epidermidis* APC 3771; APC 3761; APC 3762; APC 3763; APC 3764; APC 3766; APC 3769; APC 3770**	–	–	1	Staphyloferrin	1	–	–	–
	***S. epidermidis* APC 3768; APC 3771; APC 3773**	–	–	–	Staphyloferrin	1	–	–	–
**H2**	***S. aureus* APC 3774**	Class IId		–	2	Aureusimine	–	–	Terpene
	***S. epidermidis* APC 3810**	Class IA; Class IId		1	Staphyloferrin	1	–	–	–
	***E. faecalis* APC 3825**	Class IB; Class IIb		–	–	–	–	–	–
	***S. epidermidis* APC 3775**	Class IA; Class IId; Sactibiotic	Class IA; Class IId	–	Staphyloferrin	1	–	–	–
**H3**	***E. faecium* APC 3827**	Class IIa; Class IIc		–	–	–	–	–	–
	***S. epidermidis* APC 3776; APC 3777**	–	–	1	Staphyloferrin	1	–	–	–
**H4**	***S. epidermidis* APC 3778**	–	Class IIc	–	Staphyloferrin	1	–	–	–
	***S. epidermidis* APC 3779**	–	Class IIb	–	Staphyloferrin	1	–	–	–
**H5**	***E. faecium* APC 3828; APC 3880**	Class IIa; Class IIc		–	–	–	–	–	–
	***S. epidermidis* APC 3780**	–		1	Staphyloferrin	1	–	–	–
	***S. aureus* APC 3821; APC 3881**	–		–	2	Aureusimine	–	–	Terpene
	***S. aureus* APC 3829**	Class IIc		–	2	Aureusimine	–	–	Terpene
	***S. epidermidis* APC 3882**	Sactibiotic	–	1	Staphyloferrin	1	–	–	–
**H6**	***S. aureus* APC 3822**	Class IIc		–	2	Aureusimine	–	–	Terpene
	***S. epidermidis* APC 3787; APC 3788; APC 3789; APC 3790; APC 3791**	–		–	Staphyloferrin	1	–	–	–
**H7**	***S. epidermidis* APC 3792; APC 3793**	–		1	Staphyloferrin	1	–	–	–
	***S. hominis APC 3824***	Class IB		–	1	–	–	1	-
	***S. epidermidis* APC 3794**	Sactibiotic	–	1	Staphyloferrin	1	–	–	–
**H8**	***S. epidermidis* APC 3781; APC 3795; APC 3798**	–		1	Staphyloferrin	1	–	–	–
	***S. epidermidis* APC 3796; APC 3797; APC 3883**	Sactibiotic	–	1	Staphyloferrin	1	–	–	–
**H9**	***S. epidermidis* APC 3799; APC 3800; APC 3803**	–		1	Staphyloferrin	1	–	–	–
	***S. epidermidis* APC 3801; APC 3802**	–	Class IIb	–	Staphyloferrin	1	–	–	–
**H10**	***S. epidermidis* APC 3804; APC 3805; APC 3806**	Class IId; Class IIc		1	Staphyloferrin	1	1	–	–
	***S. epidermidis* APC 3782**	Sactibiotic; Class IId; Class IIc	Class IId; Class IIc	1	Staphyloferrin	1	1	–	–
**H 11**	***S. epidermidis* APC 3783; APC 3784**	–		–	Staphyloferrin	1	–	–	–
	***S. aureus* APC 3814**	Class IIc		–	2	Aureusimine	–	–	Terpene
**H 12**	***S. epidermidis* APC 3807**	–		1	Staphyloferrin	1	1	–	–
**H 13**	***S. epidermidis* 3808**	Sactibiotic	–	1	Staphyloferrin	–	–	–	–
**H 14**	***P. protegens* APC 3760**	Putidacin L1-like	2	–	–	6	1	–	Type III PKS; Type I PKS; betalactone; pyrrolnitrin; homoserine lactone cluster; arylpolyene
**H 15**	***S. lugdunensis* APC 3758**	TOMM		–	1	3	1	–	Terpene
**H 16**	***S. aureus* APC 3812; APC 3816; APC 3823;**	Class IId		–	2	Aureusimine	–	–	Terpene
**H 17**	***S. aureus* APC 3813**	Class IIc	–	–	2	Aureusimine	–	–	Terpene
**H 18**	***S. aureus* APC 3818; APC 3884**	Class IIc	–	–	2	Aureusimine	–	–	Terpene
**H 19**	***S. aureus* APC 3885**	–		–	2	Aureusimine	–	–	Terpene
	***S. aureus* APC 3886; APC 3887**	Class IIc	–	–	2	Aureusimine	–	–	Terpene
**H 20**	***S. aureus* APC 3819**	Class IIc	–	–	2	Aureusimine	–	–	Terpene
	***E. faecium* APC 3830; APC 3831; APC 3832; APC 3833; APC 3835; APC 3837**	Class IIa; Class IIc		–	–	–	–	–	–
**H 21**	***S. aureus* APC 3820**	Class IIc; Class IId	–	1	2	Aureusimine	–	–	Terpene
**H 22**	***S. aureus* APC 3815**	Class IIc	–	1	2	Aureusimine	–	–	Terpene
**H 23**	***E. faecium* APC 3826**	Class IIa; Class IIc		–	–	–	–	–	–
**H 24**	***E. faecium* APC 3836**	Class IIa; Class IIc		–	–	–	–	–	–
**H25**	***E. faecium* APC 3827**	Class IIa; Class IIc		–	–	–	–	–	–

*The isolates are grouped per donor from whom the bacteria were isolated.*

### Bacteriocin Identification

**FIGURE 2:**
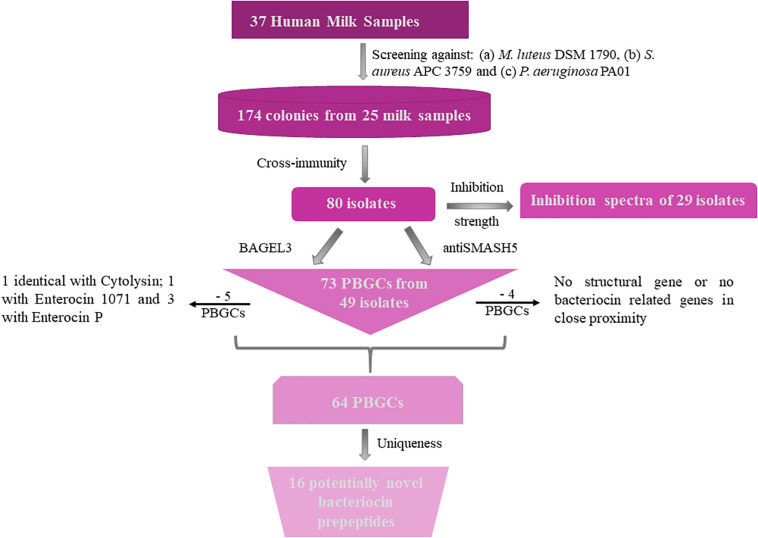
Flow chart of bacteriocin screening performed for this study. PBGC stands for Potential Bacteriocin Gene Cluster.

Bacteriocins are a divergent group of antimicrobials which utilize different systems for modification, transport, and immunity (Kotelnikova and Gelfand, 2002). We implemented an *in silico* approach to determine the diversity of the bacteriocins encoded by the 80 isolates which had a distinct immunity profile. The bacteriocin classification scheme suggested by Cotter et al. (2005, 2013) was used to distinguish between the different bacteriocin classes for Gram-positive bacteria with class I including lantibiotics and class II including the non-lantibiotics. This screen led to 49 isolates that contained 73 potential bacteriocin gene clusters (PBGCs) based on the predictions from BAGEL3 and antiSMASH5 (Table 3 and Figure 2).

Additionally, BAGEL3 identified homologs of delta-lysins in 33 of the tested isolates and antiSMASH5 found genes associated with the production of terpenes, siderophores, polyketides (PKS), and Non-Ribosomal Peptide Synthetases (NRPSs) among a plethora of secondary metabolites (Table 3). Four potential bacteriocin gene clusters were excluded due to the absence of a detectable structural gene (*S. lugdunensis* APC 3758) or essential biosynthetic-associated genes (*P. protegens* APC 3760). Of the 69 bacteriocin gene clusters identified, one was identical to the previously characterized cytolysin from *E. faecalis* (Coburn and Gilmore, 2003), one to Enterocin 1071 (Balla et al., 2000) and three to the mature peptide of Enterocin P (Cintas et al., 1997) although in some cases the leader was atypical. The remaining 64 clusters represented potentially novel bacteriocin candidates belonging to class I (*n* = 11) and class II (*n* = 53) families. From the 64 potentially novel bacteriocin gene clusters (PBGCs) identified, 16 potentially novel bacteriocin prepeptides were potentially produced by a single strain while the rest were identified in more than one isolate. A flow chart of the procedures followed is presented in Figure 2.

Similar to Egan et al. (2018), the homology of the predicted bacteriocin gene clusters to existing genes and their genetic arrangement were examined. Below we grouped PBGCs by bacteriocin class.

#### Class I Bacteriocins

Class I bacteriocins are comprised of ribosomally produced, post-translationally modified bacteriocins (RIPPs). While initially lantibiotics were the sole occupant of class I bacteriocins, nowadays it includes more than 18 subclasses (Arnison et al., 2013).

##### Lantibiotics

**FIGURE 3:**
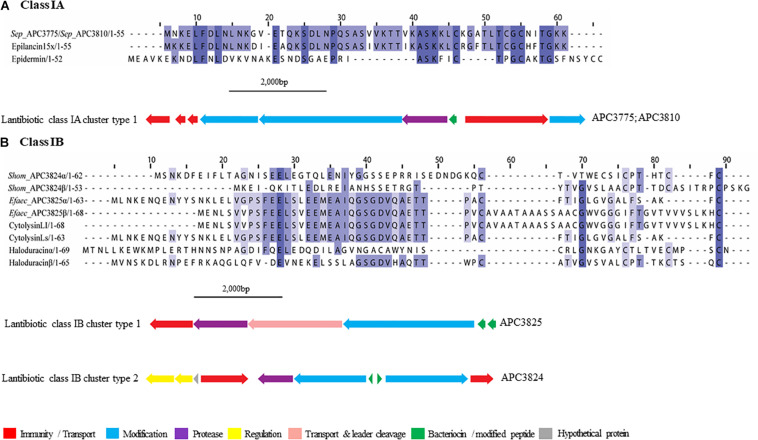
Multiple Sequence Alignments (MSA) of lantibiotic prepeptides and biosynthetic gene clusters. **(A)** Class IA MSA and predicted biosynthetic gene cluster. *Sep* stands for *S. epidermidis.* The same prepeptide was identified in *S. epidermidis* APC 3775 and APC 3810. **(B)** Class IB MSA and predicted biosynthetic gene clusters. *Shom* and *Efaec* stand for *S. hominis* and *E. faecalis* respectively.

The new lantibiotics predicted in this study (Figure 3) were grouped according to their amino acid similarity in order to facilitate comparison with previously characterized bacteriocins. Three lantibiotic gene clusters were detected in four genomes, including one classified as class IA and the remaining two as class IB (Figure 3). *S. epidermidis* APC 3775 and APC 3810 both potentially produce a class IA bacteriocin with a prepeptide displaying 81.8% similarity to epilancin 15X (Uniprot accession number: I6YXA9; Ekkelenkamp et al., 2005) and 24% to epidermin (P08136; Bierbaum et al., 1996) and contained the conserved domain PF08130 that corresponds to class IA lantibiotics.

In both of the strains encoding a class IA lantibiotic the bacteriocin modification, secretion, and immunity genes located within cluster type one (Figure 3A) exhibited strong similarities (>65%) to the bacteriocin-related genes of epilancin 15X (Elx, Velásquez et al., 2011). The cluster contained three putative immunity proteins, two modification enzymes (70.4% similarity with ElxC and 70.7% with ElxB), a protease (69.2% similarity with ElxP), an ABC transporter (80.8% similarity with ElxT) and a third modification enzyme (78.6% similarity with ElxT).

Cytolysin (Uniprot accession number: Q52052 and GenBank accession number: P08136), a two-component class IB lantibiotic, was identified in *E. faecalis* APC 3825 (IB cluster type 1; Figure 3B). An additional class IB bacteriocin gene cluster, was found in *S. hominis* APC 3824, encoding a complete class IB lantibiotic (IB, cluster type 2). In *S. hominis* APC 3824, peptide α displayed 28.8% amino acid similarity with haloduracin α while peptide β displayed 33.3% amino acid similarity to haloduracin β (Figure 3B). The operon of APC 3824 contained several lantibiotic-related genes including two LanM enzymes (conserved domain PF0145), a putative peptidase (conserved domain PF00082) and two ABC transporters containing ATP-binding subunits (conserved domain PF00005). The operon also contained a two-component regulatory system comprised of a putative histidine kinase (conserved domain PF00512) and a putative transcriptional response regulator (conserved domains PF00072 and PF00486).

##### Sactibiotics

The sactibiotics are a subgroup of Class I bacteriocins that are characterized by intramolecular bridges between cysteine sulfur and α-carbon covalent bonds (Mathur et al., 2015). These modifications are performed by radical *S*-adenosylmethionine (SAM) proteins which catalyze the formation of these thioether bonds (Rea et al., 2010).

**FIGURE 4:**
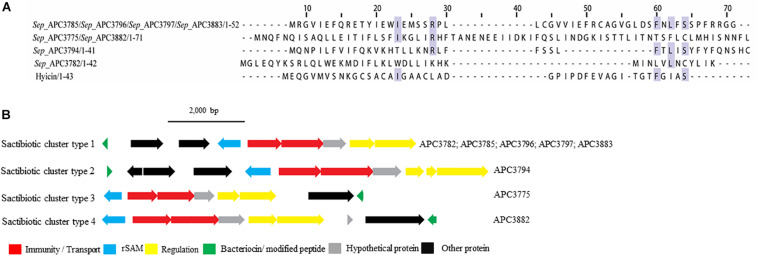
Multiple Sequence Alignments (MSA) of sactibiotic prepeptides and biosynthetic gene clusters. **(A)** MSA of the identified sactibiotic prepeptides and the known sactibiotic hyicin. *Sep* stands for *S. epidermidis.* The same prepeptide was identified in *S. epidermidis* APC 3785, APC 3796, APC 3797, and APC 3883. The same applied for the prepeptide identified in *S. epidermidis* APC 3775 and APC 3882. **(B)** Biosynthetic gene clusters of identified sactibiotics.

Four putative distinct sactibiotic peptides were predicted within eight *S. epidermidis* genomes (Figure 4A). However, when these predicted peptides were aligned with known sactibiotics, little homology was found (<20.6%).

Upstream of the gene encoding the putative bacteriocins of *S. epidermidis* APC 3785, APC 3796, APC 3797, and APC 3883 (Sactibiotics cluster type 1; Figure 4B), we detected a SAM protein (conserved domain PF04055) and an ABC transporter containing ATP-binding and permease subunits (conserved domain PF00005). The cluster also comprised a two-component regulatory system consisting of a response regulator and a histidine kinase (conserved domains PF00486, PF00072, PF00512, and PF02518, respectively). *S. epidermidis* APC 3782 had a different structural gene, even though the operon organization was similar to that of *S. epidermidis* APC 3785, APC 3796, APC 3797 and APC 3883. Downstream of the gene encoding the putative bacteriocin of *S. epidermidis* APC 3794 (Sactibiotics cluster type 2), we detected genes encoding a SAM protein (conserved domain PF04055), an ABC transporter comprised of ATP-binding (conserved domain PF00005) and permease subunits. The bacteriocin gene cluster also encoded a response regulator receiver domain, followed by a response regulator C-terminal domain and a histidine kinase. The genes encoding putative peptides of *S. epidermidis* APC 3775 (Sactibiotics cluster type 3; Figure 4B) and *S. epidermidis* APC 3882 (Sactibiotics cluster type 4; Figure 4B) were located downstream of the determinants for a histidine kinase (conserved domains PF00512; PF02518), a transcriptional response regulator (conserved domains PF00486; PF00072), an ABC transporter comprised of ATP-binding (conserved domain PF00005) and permease subunits and a SAM protein (conserved domain PF04055).

#### Class II Bacteriocins

Class II bacteriocins are defined by their unmodified nature (Eijsink et al., 2002) and their ability to permeabilize the membrane of target cells (Cotter et al., 2013). They are further divided based on the structure and activity of each class.

##### Class IIa bacteriocins

**FIGURE 5:**
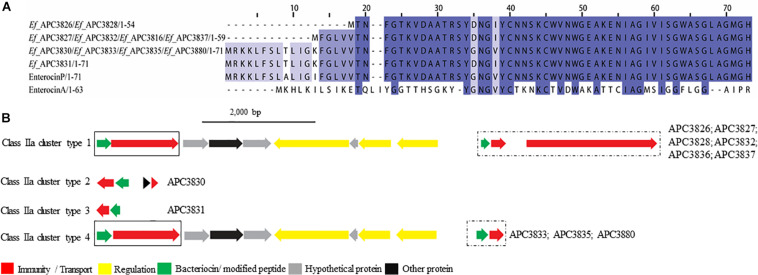
Multiple Sequence Alignments (MSA) of class IIa bacteriocin prepeptides and biosynthetic gene clusters. **(A)** MSA of the identified class IIa prepeptides and known bacteriocins Enterocin P (Uniprot accession number O30434) and Enterocin A (Uniprot accession number C8C4N1). *Ef* stands for *E. faecium.* The same prepeptide was detected in *E. faecium* APC 3826 and APC 3828. The same applied for *E. faecium* APC 3827, APC 3832, APC 3836, and APC 3837. Moreover, an identical prepeptide was found in the genomes of *E. faecium* APC 3830, APC 3833, APC 3835, and APC 3880. **(B)** Biosynthetic gene clusters of identified class IIa bacteriocins. Class IIa-related proteins are surrounded by a solix box while class IIc-related proteins are encompassed in a dashed box.

Four class IIa bacteriocins were detected by BAGEL3 in 11 *E. faecium* strains (Figure 5A) isolated from five different milk samples (Table 3). The prepeptide of *E. faecium* APC 3826 and APC 3828 displayed 94.4% similarity with Enterocin P (Uniprot accession number O30434; Cintas et al., 1997). The prepeptide identified in the genomes of *E. faecium* APC 3827, APC 3831, APC 3832, APC 3836, and APC 3837 was over 94.0% similar to Enterocin P while, *E. faecium* APC 3830, APC 3833, APC 3835, and APC 3880 had 100% identity to the mature peptide of Enterocin P, although the leader was different to Enterocin P by two amino acids (Figure 5A).

When the putative bacteriocin gene clusters were further assessed, four different gene clusters were obvious (Figure 5B). *E. faecium* APC 3826, 3827, 3828, 3832, 3836, and 3837 (IIa cluster type 1) belonged to the same bacteriocin gene cluster which contained two genes including a gene encoding a putative bacteriocin (conserved domain PF01721). The cluster also consisted of a gene encoding an immunity protein (Uniprot accession number O30435; 98.9% identity to EntP immunity protein). *E. faecium* APC 3830 was the sole isolate encoding a class IIa cluster type 2 (Figure 5B) that comprised two genes encoding bacteriocin-related proteins namely, immunity proteins. One of them was located downstream of the prepeptide (98.9% similarity to EntP immunity protein) while the second one (100% identity to EntP immunity protein) was located upstream of the structural gene (conserved domain PF01721). The bacteriocin gene cluster of *E. faecium* APC 3831 (IIa cluster type 3; Figure 5B) consisted of two bacteriocin-related genes. Class IIa cluster type 3 comprised of an immunity protein (98.9% identity to immunity protein of EntP) located downstream of the structural gene (Figure 5B). *E. faecium* APC 3833, APC 3835, and APC 3880 encode the same gene cluster (IIa cluster type 4; Figure 5B) which is comparable to cluster type I apart from the absence of the gene encoding an ABC transporter.

##### Class IIb bacteriocins

**FIGURE 6:**
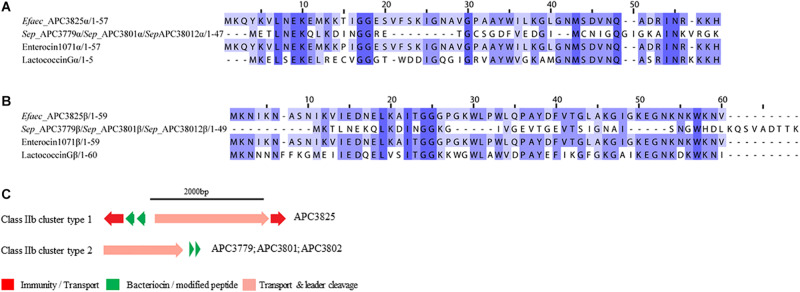
Multiple Sequence Alignments (MSA) of class IIb bacteriocin prepeptides and biosynthetic gene clusters. **(A)** MSA of the identified class IIb α prepeptides and known bacteriocins Enterocin 1071 (Uniprot accession number for α: Q9L9U6) and Lactococcin G (Uniprot accession number for α: C5IHS6). **(B)** MSA of the identified class IIb β prepeptides and known bacteriocins Enterocin 1071 (Uniprot accession number for β: Q9L9U5) and Lactococcin G (Uniprot accession number for β: C5IHS7). *Efaec* stands for *E. faecalis* and *Sep* corresponds to *S. epidermidis.* The same prepeptides were identified in *S. epidermidis* APC 3779, APC 3801, and APC 3802. **(C)** Biosynthetic gene clusters of identified class IIb bacteriocins.

Class IIb is comprised of two-peptide bacteriocins where the optimal antimicrobial activity requires the synergistic activity of both peptides (Nissen-Meyer et al., 2010). Two class IIb bacteriocin clusters were identified by BAGEL3 and antiSMASH5 (Figures 6A,B). BAGEL3 identified a bacteriocin gene cluster in *E. faecalis* APC 3825 where the prepeptide is 100% identical to Enterocin 1071 (Uniprot accession numbers: Q9L9U6 for Enterocin 1071A and Q9L9U5 for Enterocin 1071B; Balla et al., 2000). A further class IIb operon was identified by antiSMASH5 in three *S. epidermidis* strains which were isolated from two milk samples (Table 3). The α prepeptide identified in *S. epidermidis* APC 3779, APC 3801, and APC 3802 was 36.4% similar to Lactococcin Gα (Uniprot accession number C5IHS6; Moll et al., 1996; Figure 6A) while the β prepeptide displayed 24.0% identity with Lactococccin Gβ (Uniprot accession number C5IHS7; Figure 6A; Moll et al., 1996; Figure 6B).

The bacteriocin gene cluster identified in *E. faecalis* APC 3825 displayed similar genetic organization to Enterocin 1071 (Balla and Dicks, 2005) with the putative immunity gene being 39.8% similar to the immunity protein of Lactococcin G and located downstream of the structural α gene (conserved domains PF08129 and PF10439). The cluster also contained genes encoding an ABC transporter responsible for export and cleavage of the bacteriocin (conserved domains PF00005 and PF03412) and an accessory protein, both located upstream of the second structural gene (conserved domains PF08129 and PF10439; Figure 6C). A second bacteriocin gene cluster was identified in three *S*. *epidermidis* isolates, namely APC 3779, APC 3801, and APC 3802. The identified bacteriocin gene cluster contained simple genetic organization with the gene encoding an ABC transporter (conserved domains PF00005 and PF03412) being located upstream of the α structural gene (Figure 6C).

##### Class IIc bacteriocins

Class IIc bacteriocins are known as circular bacteriocins because of the covalent linkage of the N- to C- termini. Apart from being highly hydrophobic, known members of class IIc bacteriocins display little homology to each other (Martin-Visscher et al., 2011). These bacteriocins act by targeting the cytoplasmic membrane leading to pore formation and eventual cell death (Perez et al., 2018).

**FIGURE 7:**
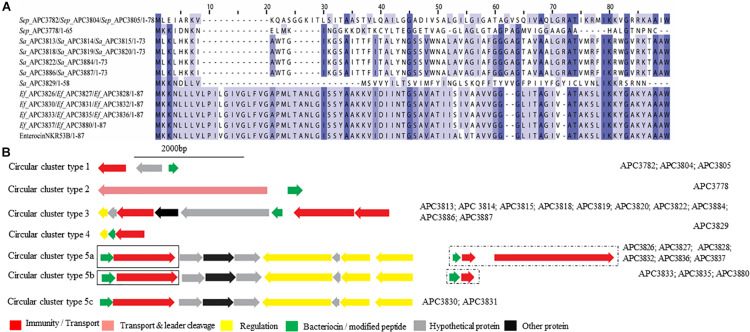
Multiple Sequence Alignments (MSA) of class IIc bacteriocin prepeptides and biosynthetic gene clusters. **(A)** MSA of the identified class IIc prepeptides and known bacteriocin EnterocinNKR-5-3B (Uniprot accession number A0A0P0YL94). *Sep* corresponds to *S. epidermidis*, *Sa* stands for *S. aureus* and *Ef* for *E. faecium.* The same prepeptide was identified in *S. epidermidis* APC 3782, APC 3804, and APC 3805. The same prepeptide was detected in *S. aureus* APC 3813, APC 3814, APC 3815, APC 3818, APC 3819, APC 3820, APC 3822, APC 3884, APC 3886, and APC 3887. Also, identical prepeptide was found in the genomes of *E. faecium* APC 3826, APC 3827, APC 3828, APC 3830, APC 3831, APC 3832, APC 3833, APC 3835, APC 3836, APC 3837, and APC 3880. **(B)** Biosynthetic gene clusters of identified class IIc bacteriocins. Class IIc-related proteins are surrounded by a dashed box while class IIa-related proteins are encompassed in a solid box.

Five potential class IIc bacteriocins (NCBI conserved domain TIGR03651) were detected (Figure 7A). More specifically, one potential class IIc bacteriocin was found in three *S. epidermidis* strains (APC 3782, APC 3804, and APC 3805) which were isolated from the same milk sample (Table 3) and displayed only 30.8% similarity to the circular bacteriocin Enterocin NKR-5-3B (Himeno et al., 2015; Uniprot accession number A0A0P0YL94). Moreover, a second putative class IIc bacteriocin was found in *S. epidermidis* APC 3778 with the prepeptide being 26.2% similar to Enterocin NKR-5-3B (Figure 7A). A third class IIc bacteriocin was detected in ten *S. aureus* strains (APC 3813, APC 3814, APC 3815, APC 3818, APC 3819, APC 3820, APC 3822, APC 3884, APC 3886, APC 3887) isolated from eight milk samples (Table 3) and the detected prepeptide was 30.1% similar to Enterocin NKR-5-3B (Uniprot accession number A0A0P0YL94). Furthermore, a fourth class IIc prepeptide was detected in *S. aureus* APC 3829 which exhibited 24.2% similarity to Enterocin NKR-5-3B (Figure 7A). Finally, a fifth class IIc prepeptide was found in 11 *E. faecium* strains (APC 3826, APC 3827, APC 3828, APC 3830, APC 3831, APC 3832, APC 3833, APC 3835, APC 3836, APC 3837, and APC 3880) isolated from six milk samples (Table 3) with the prepeptide sharing 95.4% similarity to Enterocin NKR-5-3B (Uniprot accession number A0A0P0YL94).

Five putative bacteriocin gene clusters for circular bacteriocins were identified (Figure 7B). Class IIc cluster type 1 is comprised of the three *S. epidermidis* strains (APC 3782, APC 3804, and APC 3805) and exhibits a simple genetic organization. The cluster was comprised of a gene encoding an ABC transporter containing an ATP-binding domain (conserved domain PF00005) located upstream of the putative bacteriocin (Figure 7B). *S. epidermidis* APC 3778 contained a bacteriocin gene cluster (Class IIc cluster type 2; Figure 7B) which consisted of a gene encoding an ABC transporter with a peptidase domain for cleaving the leader (conserved domains PF00005 and PF03412) located upstream of the putative bacteriocin. All ten *S. aureus* strains had the same organization in the bacteriocin gene cluster (IIc cluster type 3) which contained several bacteriocin-related genes including a transcriptional regulator and an ABC transporter containing an ATP-binding subunit. The bacteriocin gene cluster also consisted of genes encoding an ABC transporter composed of ATP-binding and permease subunits (conserved domain PF00005) located upstream of the putative structural gene (Figure 7B). *S. aureus* APC 3829 encoded a bacteriocin gene cluster (class IIc cluster type 4) which contained a transcriptional regulator and an ABC transporter (conserved domain PF00005) flanking the structural gene (Figure 7B). Within the 11 *E. faecium* strains, sharing identical structural genes, three subclasses were defined based on the organization of the gene clusters. All sub-clusters comprised of genes encoding an ABC transporter that contained ATP-binding and permease subunits (conserved domain PF00005). The sub-clusters were also composed of genes encoding a putative histidine kinase, a putative accessory protein regulator B gene and a response regulator transcription factor. The transcription factors are implicated in bacteriocin regulation, although it is not yet known to which of the two (class IIc or class IIa) bacteriocins they belong (class IIc cluster type 5a-5c; Figure 7B).

##### Class IId bacteriocins

Class IId bacteriocins are linear, single peptide bacteriocins that display no homology to class IIa bacteriocins (Cotter et al., 2013). One member of this class is Lactococcin 972, which is encoded as a 91-amino-acid prepeptide that includes a 25-amino-acid sec-dependent signal peptide and the final mature peptide (66 amino acids) (Martínez et al., 1999). Lactococcin 972 inhibits the growth of susceptible cells by interfering with septum formation (Martínez et al., 2000).

**FIGURE 8:**
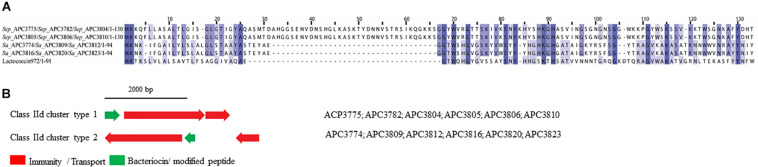
Multiple Sequence Alignments (MSA) of class IId bacteriocin prepeptides and biosynthetic gene clusters. **(A)** MSA of the identified class IId prepeptides and known bacteriocin Lactococcin 972 (Uniprot accession number O86283). *Sep* corresponds to *S. epidermidis* and *Sa* stands for *S. aureus.* The same prepeptide was identified in *S. epidermidis* APC 3775, APC 3782, APC 3804, APC 3805, APC 3806, and APC 3810. The same prepeptides were detected in *S. aureus* APC 3774, APC 3809, APC 3812, APC 3813, APC 3816, APC 3820, and APC 3823. **(B)** Biosynthetic gene clusters of identified class IId bacteriocins.

Two class IId prepeptides were identified in the study with the first being detected in six *S. epidermidis* strains identified from two human milk samples and displayed 21.1% similarity with Lactococcin 972 (Uniprot accession number: O86283; Figure 8A). The second was found in six *S. aureus* strains which were isolated from four human milk samples and was 36.3% similar to Lactococcin 972 (Uniprot accession number: O86283; Figure 8A).

Two bacteriocin class IId gene clusters were detected in this study with one being identical in all *S. epidermidis* (class IId cluster type 1) genomes while the other one was identical in all *S*. *aureus* genomes (class IId cluster type 2; Figure 8B). Class IId cluster type 1 displayed a simple genetic organization with genes encoding an ABC transporter containing a permease and an ATP-binding subunit (conserved domain PF00005), being located downstream of the structural gene (Figure 8B). Class IId cluster type 2 on the other hand, exhibited a different genetic organization and contained several bacteriocin-related genes including a putative immunity gene upstream of the structural gene and an ABC transporter containing a permease domain (conserved domain PF00005).

## Discussion

This study aimed to characterize bacteriocin producing strains isolated from healthy milk samples, combining both *in silico* and *in vitro* screening methods. This combinatorial approach enabled a more comprehensive estimation of bacteriocin production associated with human breast milk. Nevertheless, bacteriocin production can be a highly regulated process with strains requiring specific conditions and environments to induce production of these antimicrobials (Diep et al., 2000; Maldonado-Barragan et al., 2013). Therefore, the use of BAGEL3 and antiSMASH5 for bacteriocin screening facilitated the identification of bacteriocin operons in the 80 isolates with distinct immune profiles without the drawbacks and constraints of *in vitro* screening.

We screened strains isolated from the milk of 37 asymptomatic donors and found that 25 donors (67.6%) harbored strains with antimicrobial activity (Table 3) under the conditions tested. The isolated strains from the milk could have originated from the mother’s skin, the infant’s oral cavity, and/or the entero-mammary pathway (Angelopoulou et al., 2018), thus we cannot be certain about the source. During the initial screening, we observed activity against *S. aureus* APC 3759 and/or *M. luteus* DSM 1790. However, none of the strains exhibited activity against *P. aeruginosa* PA01 which is not unexpected, as bacteriocins produced by Gram-positive bacteria generally do not inhibit Gram-negative bacteria (Nikaido and Vaara, 1985; Nikaido, 2003). The majority of the bacterial species identified in this study encoding bacteriocin gene clusters are also known to encode virulence factors (Giormezis et al., 2015; Chessa et al., 2016; Madsen et al., 2017). Moreover, *S. aureus* and *S. epidermidis* are known as opportunistic pathogens in human mastitis (Angelopoulou et al., 2018). In addition, *Staphylococcus* and *Pseudomonas* belong to the healthy core milk microbiota (Boix-Amorós et al., 2016; Padilha et al., 2019). Among the isolated bacteria with high antimicrobial activity, it is notable the almost complete absence of members of the LAB group, with either high antimicrobial activity or encoding potential bacteriocins.

Cross-immunity screening resulted in the identification of 80 isolates with distinct profiles. Interestingly, strains *S. epidermidis* APC 3761, APC 3769, and APC 3772 came from the same donor (Table 3) yet they exhibited different cross-immunity profiles. However, these strains, also demonstrated immunity to the bacteriocin(s) produced by others of the same species, suggesting that these three strains might be distinct. In contrast, *S. epidermidis* APC 3765 and *S. epidermidis* APC 3790 demonstrated identical cross-immunity profiles even though they originated from different donors. This may be explained by the fact that different people harbor bacteria that produce the same bacteriocin(s). A similar situation was observed for *E. faecium* APC 3828, APC 3833, APC 3834, *Lactobacillus gasseri* APC 3841 and *C. kroppenstedtii* APC 3845, which exhibited identical immunity profiles though they originated from different donors and were produced by different species. We observed the same phenomena with other strains (Supplementary Table S1).

The combined use of BAGEL3 and antiSMASH5 in conjunction with the applied criteria led to the identification of 64 novel bacteriocin gene clusters. Among them, 16 (25%) novel bacteriocins were found while the remaining 48 bacteriocin gene clusters were identified in more than one isolate. The identified peptides belonged to all bacteriocin classes and included two lantibiotics, four sactibiotics, three class IIa, one class IIb, four circular (class IIc) and two class IId bacteriocins. This confirms that human milk is a rich source of bacteriocin producing strains. The identified bacteriocins targeted major human and bovine mastitis pathogens such as *S. aureus*, *C. tuberculostearicum* and *St. agalactiae* implying that breast milk microbiota could potentially play a beneficial role in the mammary health of lactating women by preventing breast infections and inflammation (Hunt et al., 2011). Some of the bacteriocin producing strains also potentially produce toxins. In contrast, bacteriocin producers have the potential to alter the intestinal ecology (Murphy et al., 2013), this may apply to the mammary gland of lactating women and could also influence the infant gastrointestinal tract (GIT) microbiota and the endogenous immune system, reinforcing the benefits of continued breastfeeding.

In the majority of the genomes (*n* = 66), antiSMASH5 identified secondary metabolites with potential antimicrobial activities, among others siderophores (Kohira et al., 2016), NRPSs including a lugdunin producer (a recently found thiazolidine-containing cyclic peptide antibiotic that prohibits colonization by *S. aureus*) (Zipperer et al., 2016), PKS (Zhang et al., 2014; Li et al., 2018), terpenes (Gallucci et al., 2009; Zengin and Baysal, 2014), and pyrrolinitrin (el-Banna and Winkelmann, 1998). In 32 of the isolates, BAGEL3 also identified delta-lysins, which are members of the hemolytic polypeptide family toxins produced by staphylococci and known for their antimicrobial activity (Al-Mahrous et al., 2010). However, little is known about the regulation of the production of the aforementioned secondary metabolites in natural environments such as human milk.

Our study identified three lantibiotic gene clusters, one class IA and two class IB, from four isolates. Lantibiotics are small peptides that contain internal thioether bridges due to the interaction of dehydroalanine (Dha) or dehydrobutyrine (Dhb) with intrapeptide cysteines, resulting in the formation of (β-methyl)lanthionine residues (Lan/MeLan; McAuliffe et al., 2000). The formation of Lan/MeLan is catalyzed by LanB and LanC in class IA lantibiotics such as nisin with LanB catalyzing the dehydration of amino acids and LanC catalyzing thioether formation (Marsh et al., 2010). The biosynthesis of class IB lantibiotics is catalyzed by a single LanM enzyme with both dehydratase and cyclase activity. Class IA prepeptides have an FNLD conserved region at positions −15 and −20 and a proline at −2 from the cleavage site. These play an important role in peptide modifications (Lubelski et al., 2008). The LanA identified in *S. epidermidis* APC 3775 and APC 3810 possessed an FDLN motif with the proline at −2 similar to epilancin 15X (Ekkelenkamp et al., 2005).

Four sactibiotics were identified in our study with no similarities to previously characterized peptides. The *in silico* mining identified a histidine kinase and a response regulator, suggesting that the bacteriocins are subjected to a two-component regulatory system, previously reported in bovine non-*aureus* staphylococci (Carson et al., 2017).

Despite the fact that the majority of the identified bacteriocin gene clusters were class II, only ten prepeptides were unique. We identified three novel class IIa bacteriocins in 11 *E. faecium* isolates. The novel prepeptides contained a highly conserved consensus sequence in the N-terminus of YDNGI motif instead of the “pediocin box” YGNGV which is typical of the class (Drider et al., 2006). This is the first time that a different pediocin-box has been reported with the already known class IIa bacteriocins having a highly conserved pediocin box except where V can be occasionally replaced by L (Cui et al., 2012). *E. faecium* APC 3830, APC 3833, APC 3835, and APC 3880 have the potential to produce the same class IIa bacteriocin. The core peptide is 100% homologous to enterocin P though the signal peptide is somewhat different (two amino acid difference). The different leader could impact the optimal secretion of the bacteriocin and the maturation of the pre-bacteriocin by the ABC transporter as was demonstrated by Aucher et al. (2005). In that study, site-directed mutagenesis of the leader sequence of mesentericin Y105 revealed that the conserved hydrophobic amino acids and the C-terminal GG doublet of the leader are of critical importance for the secretion and maturation of the bacteriocin.

Four potential circular (class IIc) bacteriocins were identified with similarities to Enterocin NKR-5-3B, with the majority of the circular bacteriocins in staphylococci being detected in *S. aureus* according to the existing literature (Bastos et al., 2009). Here, we identified putative novel circular bacteriocins from *S. epidermidis, S. aureus*, and *E. faecium* which displayed the highest similarity to Enterocin NKR-5-3B. All of the detected putative prepeptides contained the conserved domain linked with the circularin A/uberolysin family (TIGR03651). To date, there are only 14 circular bacteriocins that have been characterized (Perez et al., 2018) and much of the biosynthetic mechanisms including the circularization process as well as the regulatory mechanisms for production remain largely unknown. This study provides an additional four novel members (an increase of ∼25%) that may help to further elucidate the biosynthesis of this intriguing class of bacteriocins.

Two class IId bacteriocins which are linear non-pediocin like bacteriocins were found in this study. The two class IId clusters exhibited some degree of similarity with each other and at the same time both of them contained the lactococcin 972 conserved domain. Usually bacteriocins similar to lactococcin display a narrow spectrum activity against *Lactococcus* mainly because of the manner in which they bind to receptors (Kjos et al., 2009). However, this was not the case here as the detected strains exhibited wider inhibition spectra and displayed activity against a wide variety of the tested indicators. For example, *S. aureus* APC 3774 was active against *L. monocytogenes, E. faecalis, S. aureus*, and *M. luteus* among others which could potentially suggest the production of additional antimicrobials.

We identified 16 novel biosynthetic clusters belonging to all bacteriocin sub-classes across 25 human milk samples. The latter suggests not only a high degree of diversity of bacteriocins in human milk but also that it can be regarded as a promising source of novel metabolites including bacteriocins with potential implications in host defense and immune modulatory effects. Moreover, this study highlights the importance of a targeted approach to discover new effective antimicrobials that could potentially be applied to maintain good health in humans. It is anticipated that over the next few years, the number of sequenced genomes will drastically increase due to the widespread use of Next-Generation Sequencing (NGS) and the lower cost of Whole Genome Sequencing (WGS) which is empowered by third generation sequencing technologies, such as PacBio (Rhoads and Au, 2015) and Oxford Nanopore (Lu et al., 2016). With this expected proliferation in genome sequence data, there is equally a need for phenotypic trait surveying of strains such as described here to gain a more comprehensive understanding of bacteriocin-mediated interactions among coexisting species in natural communities such as human milk. The latter suggests that an increasing experimental effort to gain a more comprehensive understanding of bacteriocin mediated interactions among coexisting bacterial species in natural communities is warranted.

## Data Availability Statement

The datasets generated for this study were submitted to GenBank under BioProject number PRJNA521309.

## Ethics Statement

The studies involving human participants were reviewed and approved by the Cork Clinical Research Ethics Committee. The patients/participants provided their written informed consent to participate in this study.

## Author Contributions

AA performed the experiments and drafted the manuscript. AW advised on the screening and critically reviewed the manuscript. SS assembled the genomes and critically reviewed the manuscript. AS assembled the genomes of *P. protegens* APC 3760 and *S. lugdunensis* APC 3758, uploaded the genomes to NCBI, and critically reviewed the manuscript. PO’C, DF, LD, CS, and CH critically reviewed the manuscript. RR conceived the original idea and critically reviewed the manuscript.

## Conflict of Interest

The authors declare that the research was conducted in the absence of any commercial or financial relationships that could be construed as a potential conflict of interest.

## Footnotes

1 http://www.ncbi.nlm.nih.gov/BLAST/

2 http://www.bioinformatics.babraham.ac.uk/projects/fastqc/

## Supplementary Material

TABLE S1 Cross-immunity assays using overnight cultures from all the antimicrobial-producing strains tested against themselves and each other, with the list of antimicrobial-producing isolates down the left-hand side and the indicator strains across the top of the table. The dark green box represents inhibition of the indicator strain grown in TSB indicating that these strains might be different or sensitive to each other while light green box represents hazy zones. The yellow box represents inhibition of the indicator strain grown in MH indicating that these strains might be different or sensitive to each other while light yellow represents hazy zones. Dark blue box represents inhibition of the indicator strain grown in both conditions while light blue represents hazy zones. White boxes represent no inhibition, revealing target strains not being inhibited by the producing strain, indicating they are immune (related) to the bacteriocins produced by other producers.

## References

[B1] Abou-Dakn M. Richardt A. Schaefer-Graf U. Wöckel A. (2010). Inflammatory breast diseases during lactation: milk stasis, puerperal mastitis, abscesses of the breast, and malignant tumors – current and evidence-based strategies for diagnosis and therapy. *Breast Care.* 5 33–37. 10.1159/000272223 22619640 PMC3357165

[B2] Algburi A. Zehm S. Netrebov V. Bren A. B. Chistyakov V. Chikindas M. L. (2017). Subtilosin prevents biofilm formation by Inhibiting bacterial quorum sensing. *Probiotics Antimicrob Proteins.* 9 81–90. 10.1007/s12602-016-9242-x 27914001

[B3] Al-Mahrous M. Sandiford S. K. Tagg J. R. Upton M. (2010). Purification and characterization of a novel delta-lysin variant that inhibits *Staphylococcus aureus* and has limited hemolytic activity. *Peptides.* 31 1661–1668. 10.1016/j.peptides.2010.06.006 20561552

[B4] Altschul S. F. Gish W. Miller W. Myers E. W. Lipman D. J. (1990). Basic local alignment search tool. *J. Mol. Biol.* 215 403–410. 10.1016/S0022-2836(05)80360-2 2231712

[B5] Angelopoulou A. Field D. Ryan C. A. Stanton C. Hill C. Ross R. P. (2018). The microbiology and treatment of human mastitis. *Med. Microbiol. Immunol.* 207 83–94. 10.1007/s00430-017-0532-z 29350290

[B6] Angelopoulou A. Harris H. M. B. Warda A. K. O’Shea C.-A. Ryan C. A. Stanton C. (2020). Subclinical mastitis, a frequent but misidentified disease in lactating women. *Pediatrics* (in press).

[B7] Arnison P. G. Bibb M. J. Bierbaum G. Bowers A. A. Bugni T. S. Bulaj G. (2013). Ribosomally synthesized and post-translationally modified peptide natural products: overview and recommendations for a universal nomenclature. *Nat. Prod. Rep.* 30 108–160. 10.1039/c2np20085f 23165928 PMC3954855

[B8] Aucher W. Lacombe C. Hequet A. Frere J. Berjeaud J. M. (2005). Influence of amino acid substitutions in the leader peptide on maturation and secretion of mesentericin Y105 by *Leuconostoc mesenteroides*. *J. Bacteriol.* 187 2218–2223. 10.1128/JB.187.6.2218-2223.2005 15743973 PMC1064045

[B9] Balla E. Dicks L. M. Du Toit M. Van Der Merwe M. J. Holzapfel W. H. (2000). Characterization and cloning of the genes encoding enterocin 1071A and enterocin 1071B, two antimicrobial peptides produced by *Enterococcus faecalis* BFE 1071. *Appl. Environ. Microbiol.* 66 1298–1304. 10.1128/aem.66.4.1298-1304.2000 10742203 PMC91984

[B10] Balla E. Dicks L. M. T. (2005). Molecular analysis of the gene cluster involved in the production and secretion of enterocins 1071A and 1071B and of the genes responsible for the replication and transfer of plasmid pEF1071. *Int. J. Food. Microbiol.* 99 33–45. 10.1016/j.ijfoodmicro.2004.08.008 15718027

[B11] Bastos M. C. Ceotto H. Coelho M. L. Nascimento J. S. (2009). Staphylococcal antimicrobial peptides: relevant properties and potential biotechnological applications. *Curr. Pharm. Biotechnol.* 10 38–61. 10.2174/138920109787048580 19149589

[B12] Biagi E. Quercia S. Aceti A. Beghetti I. Rampelli S. Turroni S. (2017). The bacterial ecosystem of mother’s milk and infant’s mouth and gut. *Front. Microbiol.* 8:1214 10.3389/fmicb.2017.01214 PMC5491547 28713343

[B13] Bierbaum G. Götz F. Peschel A. Kupke T. van de Kamp M. Sahl H.-G. (1996). The biosynthesis of the lantibiotics epidermin, gallidermin. Pep5 and epilancin K7. *Antonie van Leeuwenhoek.* 69 119–127. 10.1007/BF00399417 8775972

[B14] Blin K. Shaw S. Steinke K. Villebro R. Ziemert N. Lee S. Y. (2019). antiSMASH 5.0: updates to the secondary metabolite genome mining pipeline. *Nucleic Acids Res.* 47 W81–W87. 10.1093/nar/gkz310 31032519 PMC6602434

[B15] Boix-Amorós A. Collado M. C. Mira A. (2016). Relationship between milk microbiota, bacterial load, macronutrients, and human cells during lactation. *Front Microbiol.* 7:492 10.3389/fmicb.2016.00492 PMC4837678 27148183

[B16] Bolger A. M. Lohse M. Usadel B. (2014). Trimmomatic: a flexible trimmer for Illumina sequence data. *Bioinformatics* 30 2114–2120. 10.1093/bioinformatics/btu170 24695404 PMC4103590

[B17] Cabrera-Rubio R. Mira A. Isolauri E. Laitinen K. Salminen S. Collado M. C. (2012). The human milk microbiome changes over lactation and is shaped by maternal weight and mode of delivery. *Am. J. Clin. Nutr.* 96 544–551. 10.1017/S2040174415001397 22836031

[B18] Carson D. A. Barkema H. W. Naushad S. De Buck J. (2017). Bacteriocins of non-*aureus* staphylococci isolated from bovine milk. *Appl. Environ. Microbiol.* 83 e1015–e1017. 10.1128/AEM.01015-17 28667105 PMC5561277

[B19] Chessa D. Ganau G. Spiga L. Bulla A. Mazzarello V. Campus G. V. (2016). *Staphylococcus aureus* and *Staphylococcus epidermidis* virulence strains as causative agents of persistent infections in breast implants. *Plos One* 11:e0146668. 10.1371/journal.pone.0146668 26811915 PMC4727902

[B20] Chikindas M. L. Weeks R. Drider D. Chistyakov V. A. Dicks L. M. (2018). Functions and emerging applications of bacteriocins. *Curr. Opin. Biotechnol.* 49 23–28. 10.1016/j.copbio.2017.07.011 28787641 PMC5799035

[B21] Cintas L. M. Casaus P. Håvarstein L. S. Hernández P. E. Nes I. F. (1997). Biochemical and genetic characterization of enterocin P, a novel sec-dependent bacteriocin from *Enterococcus faecium* P13 with a broad antimicrobial spectrum. *Appl. Environ. Microbiol.* 63 4321–4330. 10.1128/aem.63.11.4321-4330.1997 9361419 PMC168752

[B22] Civardi E. Garofoli F. Tzialla C. Paolillo P. Bollani L. Stronati M. (2013). Microorganisms in human milk: lights and shadows. *J. Matern. Fetal. Neonatal Med.* 26 30–34. 10.3109/14767058.2013.829693 24059550

[B23] Coburn P. S. Gilmore M. S. (2003). The *Enterococcus faecalis* cytolysin: a novel toxin active against eukaryotic and prokaryotic cells. *Cell Microbiol.* 5 661–669. 10.1046/j.1462-5822.2003.00310.x 12969372

[B24] Collado M. C. Delgado S. Maldonado A. Rodríguez J. M. (2009). Assessment of the bacterial diversity of breast milk of healthy women by quantitative real-time PCR. *Lett. Appl. Microbiol.* 48 523–528. 10.1111/j.1472-765X.2009.02567.x 19228290

[B25] Collins F. W. J. O’Connor P. M. O’Sullivan O. Gómez-Sala B. Rea M. C. Hill C. (2017). Bacteriocin gene-trait matching across the complete *Lactobacillus* pan-genome. *Sci. Rep.* 7:3481. 10.1038/s41598-017-03339-y 28615683 PMC5471241

[B26] Cotter P. D. Hill C. Ross R. P. (2005). Bacteriocins: developing innate immunity for food. *Nat. Rev. Microbiol.* 3 777–788. 10.1038/nrmicro1273 16205711

[B27] Cotter P. D. Ross R. P. Hill C. (2013). Bacteriocins — a viable alternative to antibiotics? *Nat. Rev. Microbiol.* 11 95–105. 10.1038/nrmicro2937 23268227

[B28] Crispie F. Twomey D. Flynn J. Hill C. Ross P. Meaney W. (2005). The lantibiotic lacticin 3147 produced in a milk-based medium improves the efficacy of a bismuth-based teat seal in cattle deliberately infected with *Staphylococcus aureus*. *J. Dairy Res.* 72 159–167. 10.1017/S0022029905000816 15909681

[B29] Cui Y. Zhang C. Wang Y. Shi J. Zhang L. Ding Z. (2012). Class IIa bacteriocins: diversity and new developments. *Int. J. Mol. Sci.* 13 16668–16707. 10.3390/ijms131216668 23222636 PMC3546714

[B30] Delgado S. Garcia P. Fernandez L. Jimenez E. Rodriguez-Banos M. del Campo R. (2011). Characterization of *Staphylococcus aureus* strains involved in human and bovine mastitis. *FEMS Immunol. Med. Microbiol*. 62 225–235. 10.1111/j.1574-695X.2011.00806.x 21477005

[B31] Diep D. B. Axelsson L. Grefsli C. Nes I. F. (2000). The synthesis of the bacteriocin sakacin A is a temperature-sensitive process regulated by a pheromone peptide through a three-component regulatory system. *Microbiology* 146 2155–2160. 10.1099/00221287-146-9-2155 10974103

[B32] Drider D. Fimland G. Héchard Y. McMullen L. M. Prévost H. (2006). The continuing story of class IIa bacteriocins. *Microbiol. Mol. Biol. Rev.* 70 564–582. 10.1128/MMBR.00016-05 16760314 PMC1489543

[B33] Edgar R. C. (2004). MUSCLE: multiple sequence alignment with high accuracy and high throughput. *Nucleic Acids Res.* 32 1792–1797. 10.1093/nar/gkh340 15034147 PMC390337

[B34] Egan K. Field D. Ross R. P. Cotter P. D. Hill C. (2018). In silico prediction and exploration of potential bacteriocin gene clusters within the bacterial genus *Geobacillus*. *Front. Microbiol*. 9:2116. 10.3389/fmicb.2018.02116 30298056 PMC6160750

[B35] Eijsink V. G. Axelsson L. Diep D. B. Havarstein L. S. Holo H. Nes I. F. (2002). Production of class II bacteriocins by lactic acid bacteria; an example of biological warfare and communication. *Antonie Van Leeuwenhoek* 81 639–654. 10.1023/A:1020582211262 12448760

[B36] Ekkelenkamp M. B. Hanssen M. Danny Hsu S. T. de Jong A. Milatovic D. Verhoef J. (2005). Isolation and structural characterization of epilancin 15X, a novel lantibiotic from a clinical strain of *Staphylococcus epidermidis*. *FEBS Lett.* 579 1917–1922. 10.1016/j.febslet.2005.01.083 15792796

[B37] el-Banna N. Winkelmann G. (1998). Pyrrolnitrin from *Burkholderia cepacia*: antibiotic activity against fungi and novel activities against streptomycetes. *J. Appl. Microbiol* 85 69–78. 10.1046/j.1365-2672.1998.00473.x 9721657

[B38] Fernández L. Delgado S. Herrero H. Maldonado A. Rodríguez J. M. (2008). The bacteriocin nisin, an effective agent for the treatment of staphylococcal mastitis during lactation. *J. Hum. Lact.* 24 311–316. 10.1177/0890334408317435 18689718

[B39] Fernández L. Langa S. Martin V. Maldonado A. Jimenez E. Martin R. (2013). The human milk microbiota: origin and potential roles in health and disease. *Pharmacol. Res.* 69 1–10. 10.1016/j.phrs.2012.09.001 22974824

[B40] Gallucci M. N. Oliva M. Casero C. Dambolena J. Luna A. Zygadlo J. (2009). Antimicrobial combined action of terpenes against the food-borne microorganisms *Escherichia coli*, *Staphylococcus aureus* and *Bacillus cereus*. *Flavour Fragr. J.* 24 348–354. 10.1002/ffj.1948

[B41] Giormezis N. Kolonitsiou F. Makri A. Vogiatzi A. Christofidou M. Anastassiou E. D. (2015). Virulence factors among *Staphylococcus lugdunensis* are associated with infection sites and clonal spread. *Eur. J. Clin. Microbiol. Infect Dis.* 34 773–778. 10.1007/s10096-014-2291-8 25471196

[B42] Heikkilä M. P. Saris P. E. J. (2003). Inhibition of *Staphylococcus aureus* by the commensal bacteria of human milk. *J. Appl. Microbiol.* 95 471–478. 10.1046/j.1365-2672.2003.02002.x 12911694

[B43] Himeno K. Rosengren K. J. Inoue T. Perez R. H. Colgrave M. L. Lee H. S. (2015). Identification, characterization, and three-dimensional structure of the novel circular bacteriocin, enterocin NKR-5-3B, from *Enterococcus faecium*. *Biochemistry* 54 4863–4876. 10.1021/acs.biochem.5b00196 26174911

[B44] Holmes M. A. Zadoks R. N. (2011). Methicillin resistant *S. aureus* in human and bovine mastitis. *J. Mammary Gland. Biol. Neoplasia.* 16 373–382. 10.1007/s10911-011-9237-x 21984431

[B45] Hunt K. M. Foster J. A. Forney L. J. Schütte U. M. E. Beck D. L. Abdo Z. (2011). Characterization of the diversity and temporal stability of bacterial communities in human milk. *PLoS One* 6:e21313. 10.1371/journal.pone.0021313 21695057 PMC3117882

[B46] Janek D. Zipperer A. Kulik A. Krismer B. Peschel A. (2016). High frequency and diversity of antimicrobial activities produced by nasal *Staphylococcus* strains against bacterial competitors. *PLoS Pathog.* 12:e1005812. 10.1371/journal.ppat.1005812 27490492 PMC4973975

[B47] Jiménez E. Fernandez L. Maldonado A. Martin R. Olivares M. Xaus J. (2008). Oral administration of *Lactobacillus* strains isolated from breast milk as an alternative for the treatment of infectious mastitis during lactation. *Appl. Environ. Microbiol.* 74 4650–4655. 10.1128/AEM.02599-07 18539795 PMC2519365

[B48] Kjos M. Nes I. F. Diep D. B. (2009). Class II one-peptide bacteriocins target a phylogenetically defined subgroup of mannose phosphotransferase systems on sensitive cells. *Microbiology* 155 2949–2961. 10.1099/mic.0.030015-0 19477899

[B49] Klostermann K. Crispie F. Flynn J. Meaney W. J. Ross R. P. Hill C. (2009). Efficacy of a teat dip containing the bacteriocin lacticin 3147 to eliminate Gram-positive pathogens associated with bovine mastitis. *J. Dairy Res.* 77 231–238. 10.1017/S0022029909990239 19785910

[B50] Kohira N. West J. Ito A. Ito-Horiyama T. Nakamura R. Sato T. (2016). *In vitro* antimicrobial activity of a siderophore cephalosporin, S-649266, against *enterobacteriaceae* clinical isolates, including carbapenem-resistant strains. *Antimicrob. Agents Chemother.* 60 729–734. 10.1128/AAC.01695-15 26574013 PMC4750680

[B51] Kort R. Caspers M. van de Graaf A. van Egmond W. Keijser B. Roeselers G. (2014). Shaping the oral microbiota through intimate kissing. *Microbiome* 2:41. 10.1186/2049-2618-2-41 25408893 PMC4233210

[B52] Kotelnikova E. A. Gelfand M. S. (2002). Bacteriocin production by gram-positive bacteria and the mechanisms of transcriptional regulation. *Russ. J. Genet.* 38 628–641. 10.1023/A:1016035700012

[B53] Li R. Fein S. B. Chen J. Grummer-Strawn L. M. (2008). Why mothers stop breastfeeding: mothers’ self-reported reasons for stopping during the first year. *Pediatrics* 122 S69–S76. 10.1542/peds.2008-1315i 18829834

[B54] Li Y. Li Y. Li Q. Gao J. Wang J. Luo Y. (2018). Biosynthetic and antimicrobial potential of actinobacteria isolated from bulrush rhizospheres habitat in Zhalong Wetland. *China. Arch. Microbiol.* 200 695–705. 10.1007/s00203-018-1474-6 29368168

[B55] Lu H. Giordano F. Ning Z. (2016). Oxford nanopore MinION sequencing and genome assembly. *Genomics Proteomics Bioinformatics* 14 265–279. 10.1016/j.gpb.2016.05.004 27646134 PMC5093776

[B56] Lubelski J. Rink R. Khusainov R. Moll G. N. Kuipers O. P. (2008). Biosynthesis, immunity, regulation, mode of action and engineering of the model lantibiotic nisin. *Cell Mol. Life. Sci.* 65 455–476. 10.1007/s00018-007-7171-2 17965835 PMC11131864

[B57] Madsen K. T. Skov M. N. Gill S. Kemp M. (2017). Virulence factors associated with *Enterococcus faecalis* infective endocarditis: a mini review. *Open Microbiol J.* 11 1–11. 10.2174/1874285801711010001 28567146 PMC5418949

[B58] Maldonado-Barragan A. Caballero-Guerrero B. Lucena-Padros H. Ruiz-Barba J. L. (2013). Induction of bacteriocin production by coculture is widespread among plantaricin-producing *Lactobacillus plantarum* strains with different regulatory operons. *Food Microbiol.* 33 40–47. 10.1016/j.fm.2012.08.009 23122499

[B59] Marsh A. J. O’Sullivan O. Ross R. P. Cotter P. D. Hill C. (2010). *In silico* analysis highlights the frequency and diversity of type 1 lantibiotic gene clusters in genome sequenced bacteria. *BMC Genomics* 11:679. 10.1186/1471-2164-11-679 21118552 PMC3091789

[B60] Martin C. R. Ling P.-R. Blackburn G. L. (2016). Review of infant feeding: key features of breast milk and infant formula. *Nutrients.* 8:279. 10.3390/nu8050279 27187450 PMC4882692

[B61] Martin M. (2011). Cutadapt removes adapter sequences from high-throughput sequencing reads. *EMBnet J.* 17 10–12. 10.14806/ej.17.1.200

[B62] Martínez B. Fernández M. Suárez E. J. Rodríguez A. (1999). Synthesis of lactococcin 972, a bacteriocin produced by *Lactococcus lactis* IPLA 972, depends on the expression of a plasmid-encoded bicistronic operon. *Microbiology* 145 3155–3161. 10.1099/00221287-145-11-3155 10589723

[B63] Martínez B. Rodriguez A. Suárez J. E. (2000). Lactococcin 972, a bacteriocin that inhibits septum formation in lactococci. *Microbiology* 146 949–955. 10.1099/00221287-146-4-949 10784053

[B64] Martin-Visscher L. A. van Belkum M. J. Vederas J. C. (2011). “Class IIc or circular bacteriocins,” in *Prokaryotic Antimicrobial Peptides: From Genes to Applications*, eds Drider D. Rebuffat S. (New York, NY: Springer), 213–236. 10.1007/978-1-4419-7692-5_12

[B65] Mathur H. Rea M. C. Cotter P. D. Hill C. Ross R. P. (2015). The sactibiotic subclass of bacteriocins: an update. *Curr. Protein Pept. Sci.* 16 549–558. 10.2174/1389203716666150515124831 26031307

[B66] McAuliffe O. Hill C. Ross R. P. (2000). Each peptide of the two-component lantibiotic lacticin 3147 requires a separate modification enzyme for activity. *Microbiology* 146 2147–2154. 10.1099/00221287-146-9-2147 10974102

[B67] Mediano P. Fernández L. Jiménez E. Arroyo R. Espinosa-Martos I. Rodríguez J. M. (2017). Microbial diversity in milk of women with mastitis: potential role of coagulase-negative staphylococci, viridans group streptococci, and corynebacteria. *J. Hum. Lact.* 33 309–318. 10.1177/0890334417692968 28418794

[B68] Moll G. Ubbink-Kok T. Hildeng-Hauge H. Nissen-Meyer J. Nes I. F. Konings W. N. (1996). Lactococcin G is a potassium ion-conducting, two-component bacteriocin. *J. Bacteriol.* 178:600. 10.1128/jb.178.3.600-605.1996 8550488 PMC177700

[B69] Murphy E. F. Cotter P. D. Hogan A. O’Sullivan O. Joyce A. Fouhy F. (2013). Divergent metabolic outcomes arising from targeted manipulation of the gut microbiota in diet-induced obesity. *Gut.* 62 220–226. 10.1136/gutjnl-2011-300705 22345653

[B70] Nikaido H. (2003). Molecular basis of bacterial outer membrane permeability revisited. *Microbiol. Mol. Biol. Rev.* 67 593–656. 10.1128/mmbr.67.4.593-656.2003 14665678 PMC309051

[B71] Nikaido H. Vaara M. (1985). Molecular basis of bacterial outer membrane permeability. *Microbiol. Rev.* 49 1–32. 10.1128/mmbr.49.1.1-32.1985 2580220 PMC373015

[B72] Nissen-Meyer J. Oppegård C. Rogne P. Haugen H. S. Kristiansen P. E. (2010). Structure and mode-of-action of the two-peptide (class-IIb) bacteriocins. *Probiotics Antimicrob. Proteins.* 2 52–60. 10.1007/s12602-009-9021-z 20383320 PMC2850506

[B73] Nurk S. Meleshko D. Korobeynikov A. Pevzner P. A. (2017). metaSPAdes: a new versatile metagenomic assembler. *Genome Res.* 27 824–834. 10.1101/gr.213959 28298430 PMC5411777

[B74] O’Sullivan J. N. Rea M. C. O’Connor P. M. Hill C. Ross R. P. (2019). Human skin microbiota is a rich source of bacteriocin-producing staphylococci that kill human pathogens. *FEMS Microbiol. Ecol.* 95:fiy241. 10.1093/femsec/fiy241 30590567 PMC6340406

[B75] Overbeek R. Olson R. Pusch G. D. Olsen G. J. Davis J. J. Disz T. (2014). The SEED and the rapid annotation of microbial genomes using subsystems technology (RAST). *Nucleic Acids Res.* 42 D206–D214. 10.1093/nar/gkt1226 24293654 PMC3965101

[B76] Padilha M. Danneskiold-Samsoe N. B. Brejnrod A. Hoffmann C. Cabral V. P. Iaucci J. M. (2019). The human milk microbiota is modulated by maternal diet. *Microorganisms* 7 502. 10.3390/microorganisms7110502 31671720 PMC6920866

[B77] Paviour S. Musaad S. Roberts S. Taylor G. Taylor S. Shore K. (2002). *Corynebacterium* species isolated from patients with mastitis. *Clin. Infect. Dis.* 35 1434–1440. 10.1086/344463 12439810

[B78] Perez P. F. Doré J. Leclerc M. Levenez F. Benyacoub J. Serrant P. (2007). Bacterial imprinting of the neonatal immune system: lessons from maternal cells? *Pediatrics* 119:e724. 10.1542/peds.2006-1649 17332189

[B79] Perez R. H. Zendo T. Sonomoto K. (2018). Circular and leaderless bacteriocins: biosynthesis, mode of action, applications, and prospects. *Front. Microbiol.* 9:2085. 10.3389/fmicb.2018.02085 30233551 PMC6131525

[B80] Rea M. C. Sit C. S. Clayton E. Connor P. M. Whittal R. M. Zheng J. (2010). Thuricin CD, a posttranslationally modified bacteriocin with a narrow spectrum of activity against *Clostridium difficile*. *Proc. Natl. Acad. Sci. U.S.A.* 107 9352–9357. 10.1073/pnas.0913554107 20435915 PMC2889069

[B81] Rhoads A. Au K. F. (2015). PacBio sequencing and its applications. *Genom. Proteom. Bioin.* 13 278–289. 10.1016/j.gpb.2015.08.002 PMC4678779 26542840

[B82] Ross A. A. Doxey A. C. Neufeld J. D. (2017). The skin microbiome of cohabiting couples. *mSystems* 2:e043-17. 10.1128/mSystems.00043-17 28761935 PMC5527301

[B83] Shah M. M. Iihara H. Noda M. Song S. X. Nhung P. H. Ohkusu K. (2007). dnaJ gene sequence-based assay for species identification and phylogenetic grouping in the genus *Staphylococcus*. *Int. J. Syst. Evol. Microbiol.* 57 25–30. 10.1099/ijs.0.64205-0 17220435

[B84] van Heel A. J. de Jong A. Montalban-Lopez M. Kok J. Kuipers O. P. (2013). BAGEL3: Automated identification of genes encoding bacteriocins and (non-)bactericidal posttranslationally modified peptides. *Nucleic Acids Res.* 41 W448–W453. 10.1093/nar/gkt391 23677608 PMC3692055

[B85] Velásquez J. E. Zhang X. van der Donk W. A. (2011). Biosynthesis of the antimicrobial peptide epilancin 15X and its N-terminal lactate. *Chem. Biol.* 18 857–867. 10.1016/j.chembiol.2011.05.007 21802007 PMC3161514

[B86] Waterhouse A. M. Procter J. B. Martin D. M. Clamp M. Barton G. J. (2009). Jalview Version 2-a multiple sequence alignment editor and analysis workbench. *Bioinformatics* 25 1189–1191. 10.1093/bioinformatics/btp033 19151095 PMC2672624

[B87] Wayah S. B. Philip K. (2018). Pentocin MQ1: a novel, broad-spectrum, pore-forming bacteriocin from *Lactobacillus pentosus* CS2 with quorum sensing regulatory mechanism and biopreservative potential. *Front. Microbiol.* 9:564. 10.3389/fmicb.2018.00564 29636737 PMC5880951

[B88] WHO (2000). *Mastitis: Causes and Management.* Geneva: World Health Organization, 1–45.

[B89] Wong S. C. Y. Poon R. W. S. Chen J. H. K. Tse H. Lo J. Y. C. Ng T. K. (2017). *Corynebacterium kroppenstedtii* is an emerging cause of mastitis especially in patients with psychiatric illness on antipsychotic medication. *Open Forum Infect. Dis.* 4:ofx096. 10.1093/ofid/ofx096 28852671 PMC5570011

[B90] Zengin H. Baysal A. H. (2014). Antibacterial and antioxidant activity of essential oil terpenes against pathogenic and spoilage-forming bacteria and cell structure-activity relationships evaluated by SEM microscopy. *Molecules* 19 17773–17798. 10.3390/molecules191117773 25372394 PMC6272013

[B91] Zhang J. Du L. Liu F. Xu F. Hu B. Venturi V. (2014). Involvement of both PKS and NRPS in antibacterial activity in *Lysobacter enzymogenes* OH11. *FEMS Microbiol. Lett.* 355 170–176. 10.1111/1574-6968.12457 24801439 PMC4106713

[B92] Zipperer A. Konnerth M. C. Laux C. Berscheid A. Janek D. Weidenmaier C. (2016). Human commensals producing a novel antibiotic impair pathogen colonization. *Nature* 535 511–516. 10.1038/nature18634 27466123

